# Distinctive Features of the Buffer Capacity of Polyelectrolyte Microcapsules Formed on MnCO_3_ Core

**DOI:** 10.3390/polym17152149

**Published:** 2025-08-06

**Authors:** Aleksandr L. Kim, Alexey V. Dubrovskii, Sergey A. Tikhonenko

**Affiliations:** 1 Moscow Polytechnic University (Moscow Polytech), Bolshaya Semyonovskaya Str., 38, 107023 Moscow, Russia; 2 Institute of Theoretical and Experimental Biophysics Russian Academy of Science, Institutskaya St., 3, 142290 Puschino, Moscow Region, Russia; dav198@mail.ru

**Keywords:** buffer capacity, polyelectrolyte microcapsules, MnCO_3_, PMC, NaCl, Na_2_SO_4_

## Abstract

The development of layer-by-layer polyelectrolyte microcapsules (PMCs) with defined buffer capacity (BC) is a key task for creating stable systems in biomedicine and materials science. Manganese carbonate (MnCO_3_), which shares properties with CaCO_3_ and the ability to form hollow structures, represents a promising alternative. However, its interaction with polyelectrolytes and its influence on BC remain insufficiently studied. This research focuses on determining the BC of PMCs templated on MnCO_3_ cores under varying ionic strength (0.22–3 M NaCl) and temperature (60–90 °C), as well as comparing the results with PMCs templated on CaCO_3_ and PS cores. It was found that MnCO_3_-based PMCs (PMC_Mn_) exhibit hybrid behavior between CaCO_3_- and PS-based PMCs: the BC dynamics of PMC_Mn_ and CaCO_3_-based PMCs (PMC_Ca_) in water are identical. At different ionic strength at pH < 5, the BC of PMCMn and PS-based PMCs (PMC_PS_) remains unchanged, while at pH > 8.5, the BC of PMC_Mn_ increases only at 3 M NaCl. The BC of PMC_Mn_ remains stable under heating, whereas the BC of PMC_Ca_ and PMC_PS_ decreases. These results confirm that the choice of core material dictates PMC functionality, paving the way for adaptive systems in biosensing and controlled drug delivery.

## 1. Introduction

The polyelectrolyte microcapsules (PMCs) have been known as the multifunctional nanostructures fabricated via layer-by-layer (LbL) deposition of oppositely charged poly-electrolytes onto the spherical sacrificial templates, followed by core dissolution [1]. Those microcapsules have had some broad applications in medicine, food technology, and smart-materials due to their tunable physicochemical properties [1,2,3,4,5]. A critical parameter governing PMC functionality has been its buffer capacity (BC), which reflects the system’s ability to maintain the stable hydrogen ion concentrations in the solution [6,7]. That property has been essential for preserving the activity of the encapsulated molecules, such as enzymes [8,9,10], fluorescent dyes [11,12], and catalytic nanoparticles [13,14], particularly under fluctuating pH [15], ionic strength, and temperature [16,17,18,19].

Previous studies have demonstrated that BC significantly depends on the template/core type [20]. PMCs templated on polystyrene (PS) and calcium carbonate (CaCO_3_) exhibit dis-tinct BC profiles in response to ionic strength and temperature changes. CaCO_3_-based PMCs (PMCCa) display BC across a wide pH range (5.5–9), whereas PS-based PMCs (PMCPS) ex-hibit BC changes only at pH > 8. Increasing NaCl concentration gradually enhances BC in PMCPS up to 3 M, while PMCCa show maximal BC enhancement at 1 M NaCl. These differences are attributed to shell morphology: PS templates form dense polyelectrolyte layers with abundant ion pairs, whereas CaCO_3_-based PMCs possess a porous structure with channel-like pores [21,22,23].

**Figure 1:**
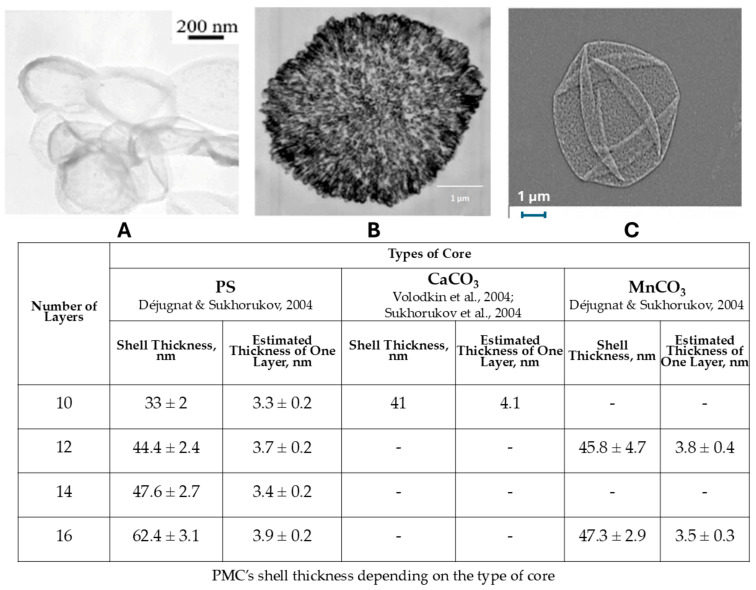
Electron microscopy image of polyelectrolyte microcapsules, formed on polystyrene [28] (**A**), CaCO_3_ [29] (**B**) and MnCO_3_ [30] (**C**). PMC’s shell thickness depending on the type of core (adapted from [23]).

However, differences in the buffer capacity (BC) may also arise from the chemical in-teractions between the template material and the polyelectrolyte shell of the polyelectrolyte microcapsules (PMCs) [20]. Therefore, this study proposes manganese carbonate (MnCO_3_) as an alternative template (core) due to its similarity to CaCO_3_ as an insoluble carbonate of a divalent cation, sharing comparable chemical properties. Additionally, MnCO_3_ enables the formation of hollow PMCs, akin to those templated on polystyrene (PS) cores [24,25]. Despite similarities to these two PMC types, capsules templated on MnCO_3_ (PMC_Mn_) may exhibit a unique BC modulation pattern. The use of MnCO_3_ cores significantly alters the assembly of polyelectrolyte layers, as evidenced by shifts in zeta potential during layer-by-layer deposi-tion, and modifies the density of proton-active amine groups in the poly(allylamine hydro-chloride) (PAH)/poly(styrene sulfonate) (PSS) shell [26]. Unlike Ca^2+^ ions, Mn^2+^ ions released during core dissolution can form stable complexes with PAH amine groups in the PMC shell [27]. This interaction affects the protonation/deprotonation equilibrium of functional groups, potentially modifying the buffering properties of the capsules. For instance, in acidic envi-ronments (pH < 5), Mn^2+^ stabilizes the positive charge of PAH via electrostatic binding, en-hancing BC, whereas under alkaline conditions (pH > 8), deprotonated amine groups form a more flexible network capable of reversible structural rearrangements [26]. Morphologically, MnCO_3_-templated PMCs differ from other capsules Figure 1.

As can be seen from Figure 1, morphologically, MnCO_3_-templated PMCs differ from other capsules, it has a linear shell thickness growth (~3.8 nm/layer). Furthermore, while PMCs templated on MnCO_3_ cores, like those formed on polystyrene cores, are hollow inside, their shell thickness differs significantly. In contrast, capsules templated on CaCO_3_ cores have an internal structure filled with a polyelectrolyte complex. Also, according to our previous review article, there was a sharp size increase in alkaline media (pH > 12.5) [23].

The aim of this study is to evaluate the buffer capacity of polyelectrolyte microcapsules synthesized on MnCO_3_ cores using acid-base titration and differential analysis (Equation (1)) and to analyze the effects of ionic strength and temperature on the buffer capacity. The research hypothesis posits that MnCO_3_-templated PMCs will combine characteristics of both CaCO_3_- and PS-based microcapsules. While prior studies have characterized BC in PMCs templated on CaCO_3_ [6] and PS [20], the role of MnCO_3_ cores remains unexplored. This work addresses this gap by systematically evaluating BC in MnCO_3_-templated PMCs under variable ionic strength and temperature, revealing hybrid behavior not reported elsewhere. The findings will advance the understanding of the core (template) role in determining the functional properties of PMCs and enable the optimization of their applications in dynamic environments, such as drug delivery or biosensors.

## 2. Materials and Methods

### 2.1. Materials

Sodium polystyrene sulfonate (PSS) and polyallylamine hydrochloride (PAH) (MW = 70 kDa, residual monomer < 10%, Sigma-Aldrich, St. Louis, MO, USA, Cat. # 243051 and 283223) served as the polyelectrolytes. Manganese(II) chloride tetrahydrate (MnCl_2_·4H_2_O, ≥99.9%, Reakhim, Moscow, Russia), sodium carbonate (Na_2_CO_3_, anhydrous, ≥99.9%, Reakhim), sodium chloride (NaCl, ≥99.9%, Reakhim, Moscow, Russia), sodium hydroxide (NaOH, pellets, ≥99.9%, Reakhim, Moscow, Russia), hydrochloric acid (HCl, 37%, ≥99.9%, Reakhim, Moscow, Russia), and ethylenediaminetetraacetic acid disodium salt dihydrate (EDTA-Na_2_, ≥99.0%, Sigma-Aldrich, St. Louis, MO, USA, Cat. # E4884) were used as received. Ultrapure water (Milli-Q, 18.2 MΩ·cm) was used for all solutions and washings.

### 2.2. Synthesis of MnCO_3_ Microtemplates

Spherical MnCO_3_ microparticles were precipitated by rapid mixing of equimolar solutions [31]. Briefly, 50 mL of 0.33 M MnCl_2_ solution was injected within 5 s into 50 mL of vigorously stirred (800 rpm) 0.33 M Na_2_CO_3_ solution at ambient temperature (23 ± 2 °C). Stirring was halted precisely 30 s post-mixing. The suspension was aged quiescently for 60 min to allow for complete particle precipitation and crystal ripening. Rapid mixing creates high supersaturation, favoring instantaneous nucleation over growth. Quiescent aging enables Oswald ripening where smaller crystals dissolve and reprecipitate onto larger ones, reducing surface energy. This yields monodisperse spheres, which are critical for uniform capsule properties. Ripening progression was monitored periodically using bright-field optical microscopy (Olympus CX23, Olympus Corporation, Tokyo, Japan). The supernatant was then decanted, and the settled microspherulites were washed three times with ultrapure water via centrifugation–resuspension cycles (10,000 rpm, 1 min). The resulting monodisperse particles exhibited an average diameter of 4.5 ± 1.0 µm, determined statistically from micrographs.

### 2.3. Fabrication of Polyelectrolyte Microcapsules (PMCs)

Hollow MnCO_3_-templated PMCs were fabricated via sequential layer-by-layer (LbL) assembly [24,32]. Washed MnCO_3_ particles were alternately exposed to 2 mg/mL solutions of the polyelectrolytes (PAH or PSS) dissolved in 0.5 M NaCl. Adsorption steps (10 min per layer) were followed by triple washing with 0.5 M NaCl solution (centrifugation at 3000 rpm for 5 min) to ensure removal of unbound polymer chains. The shell architecture consisted of a base sequence (PSS/PAH)n, terminated with an outer PAH layer. Following deposition of the desired n bilayers, the sacrificial MnCO_3_ cores were dissolved by incubating the coated particles in 0.2 M ethylenediaminetetraacetic acid (EDTA) solution (pH 7.0) for 12 h under gentle agitation. The liberated hollow capsules were purified by three cycles of centrifugation (2500 rpm, 10 min) and resuspension in ultrapure water. The final PMC diameter was 3.0 ± 0.5 µm, verified using dynamic light scattering (Zetasizer Nano ZS, Malvern Panalytical, London, UK; measurement angle 173°, temperature 25 °C).

### 2.4. Thermal Stability Assessment

PMC thermal resilience was probed by incubating suspensions in tightly sealed polypropylene tubes within a calibrated thermostatic bath (Lauda Ecoline RE 112, Lauda-Königshofen, Germany). Samples were exposed to target temperatures (60 °C or 90 °C) for 60 min. Post-incubation, suspensions were passively cooled to an ambient laboratory temperature (23 ± 1 °C) for 120 min prior to buffer capacity measurements to eliminate transient thermal effects [20].

### 2.5. Buffer Capacity (BC) Quantification

BC was assessed by acid-base titration of standardized PMC suspensions. A suspension containing 6.6 × 10^9^ capsules in 8 mL ultrapure water was titrated at 25 °C using either 0.001 M HCl or 0.005 M NaOH titrants. pH was continuously monitored with a calibrated glass electrode (HI1131B, Hanna Instruments, Smithfield, RI, USA). Titrant additions were manually controlled to induce pH shifts ≥ 0.02 units per step. The instantaneous buffer capacity (BC, mmol/L/pH unit) at each titration point (i) was computed from the differential change in the added base ($n_{NaOH}$) relative to the corresponding pH change using the central difference formula [20,33]:

(1) $$BC=\frac{n_{NaOH}\left(i+1\right)-n_{NaOH}\left(i-1\right)\;}{pH\left(i+1\right)-\;pH\left(i-1\right)},$$

where $n_{NaOH}$ is the number of moles of NaOH added and (*i* − 1) and (*i* + 1) denote the immediately preceding and succeeding titration points, respectively. This differential approach (Equation (1)) captures nonlinear BC responses to pH shifts, which is essential for quantifying how ionic strength and temperature alter protonation equilibria in the PAH/PSS shell.

### 2.6. Data Analysis and Statistics

Each experimental condition (pH, ionic strength, temperature) was independently replicated five times (n = 5). Due to inherent titration variability preventing exact pH replication across runs, BC values from all replicates were binned into 0.1 pH unit intervals across the measured range (pH 4–9). Reported BC values represent the mean ± standard deviation (SD) within each pH bin. Error bars in figures denote SD for both the mean pH (X-axis) and the mean BC (Y-axis) within each binned interval. Statistical analysis and graphing were performed using OriginPro 2024b (OriginLab Corporation, Northampton, MA, USA) [20].

## 3. Results and Discussion

The buffer capacity (BC) of polyelectrolyte microcapsules (PMCs) serves as an indicator of their ability to regulate local hydrogen proton concentrations—a critical property for preserving the structural and functional integrity of encapsulated objects. Although previous studies have established pH-dependent buffering behavior in PMCs templated on calcium carbonate (CaCO_3_) [7], literature data suggest that the chemical composition and dissolution kinetics of the core material influence electrostatic interactions, layer permeability, and ultimately, the buffering efficiency of the resulting microcapsules [26]. In this study, we shifted focus to manganese carbonate (MnCO_3_), which shares the advantages of CaCO_3_, enabling the synthesis of hollow PMCs. Unlike PMCs templated on polystyrene particles (PMCPS), MnCO_3_-based PMCs allow the encapsulation of biological molecules via coprecipitation without organic solvents or significant pH shifts that could compromise the functionality of encapsulated molecules [23,34,35].

**Figure 2:**
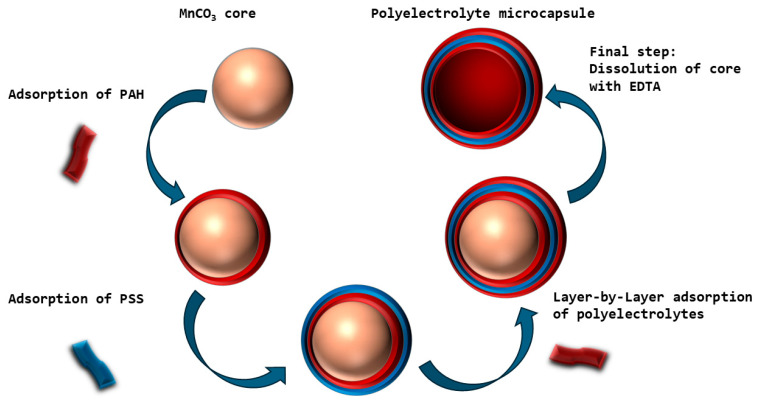
Schematic of the preparation of polyelectrolyte microcapsules on MnCO_3_ particles. The size distribution of the polyelectrolyte microcapsules (PMCs), formed on an MnCO_3_ core (abbreviated as PMC_Mn_), was subsequently determined (Figure 3).

In this work, we evaluated the BC of polyelectrolyte microcapsules synthesized on MnCO_3_ cores and analyzed the effects of ionic strength and temperature on their BC dynamics. The study employed microcapsules with a (PAH/PSS)_3_/PAH composition, fabricated via layer-by-layer adsorption of polyelectrolytes—polyallylamine (PAH) and polystyrene sulfonate (PSS)—onto MnCO_3_ particles. The MnCO_3_ core was subsequently removed through selective dissolution. The fabrication scheme of the polyelectrolyte microcapsules is illustrated in Figure 2.

**Figure 3:**
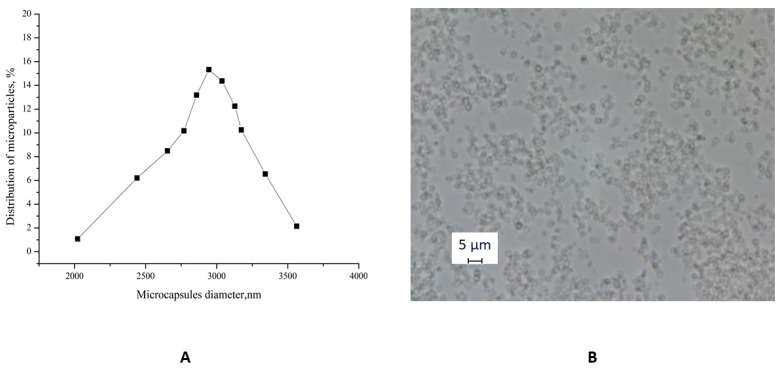
(**A**) Diameter distribution function of PMCs. (**B**) Optical microscopy images of PMCs.

The optical microscopy images of PMCs (Figure 3B) demonstrate their morphological homogeneity. The microcapsules exhibited an average diameter of 3 μm with a polydispersity index of 1.01% (Figure 3A).

**Figure 4:**
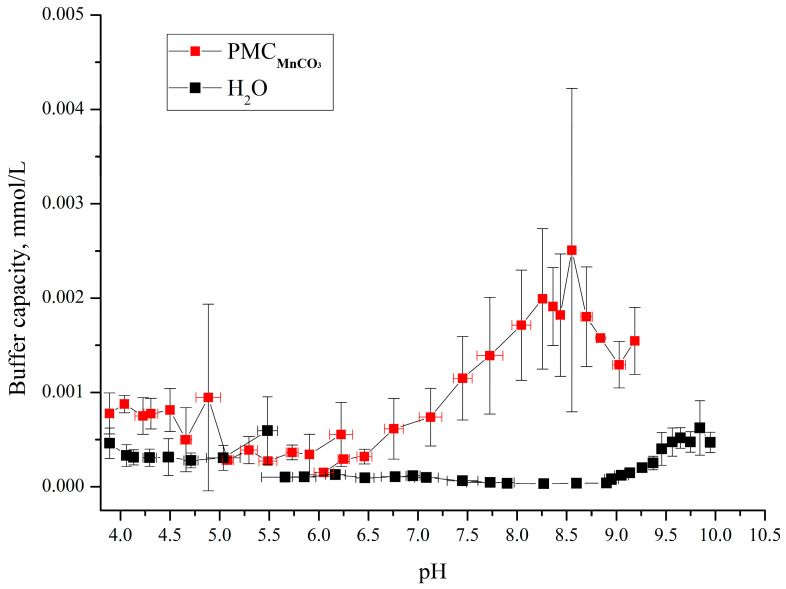
Buffer capacity of PMCs formed on MnCO_3_ with composition (PAH/PSS)_3_/PAH in the pH range from 3.5 to 10.

The synthesized polyelectrolyte microcapsules (PMCs) templated on MnCO_3_ particles (abbreviated as PMC_Mn_) were used to assess their buffer capacity (BC). For this purpose, a PMC suspension (6.7 × 10^9^ microcapsules in 8 mL of water) was titrated with acid or base across a pH range of 4–10, and the pH changes were monitored using a pH meter. The results of the BC determination for the pH range 4–10 are presented in Figure 4.

**Figure 5:**
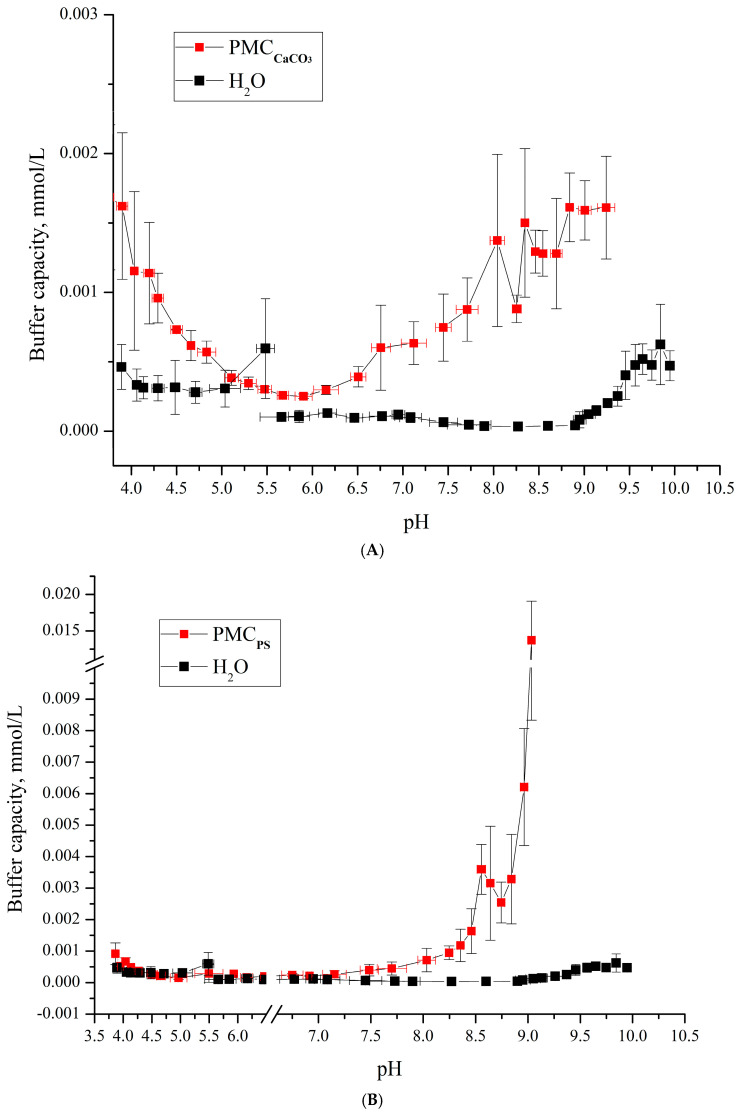
Buffer capacity of microcapsules PMC_Ca_ (PSS/PAH)_3_ and water in the pH range from 4 to 9. (**A**) PMC_CaCO3_ Adapted from [6]; (**B**) PMC_Ps_ Adapted from [20].

As shown in the figure, the buffer capacity (BC) of the microcapsules templated on MnCO_3_ particles differs from that of water at pH > 6.2 and pH < 5. In our previous work, we determined the BC of polyelectrolyte microcapsules templated on CaCO_3_ cores (abbreviated as PMCCa) [6] and polystyrene cores (PMCPS) [20]. Comparative analysis of the BC across these three capsule types revealed significant differences: PMC_Ca_ and PMC_PS_ exhibit distinct BC profiles across the entire pH range compared to PMC_Mn_. Results from our prior studies are summarized in Figure 5.

A comparison of the results presented in Figure 4 and Figure 5 reveals that PMC_Mn_ exhibits buffer capacity (BC) behavior analogous to PMCCa: BC increases at pH > 6.2 under alkaline conditions and at pH < 5 under acidic conditions. This suggests that the use of carbonate cores (e.g., MnCO_3_ and CaCO_3_) leads to a consistent model of BC dynamics.

**Figure 6:**
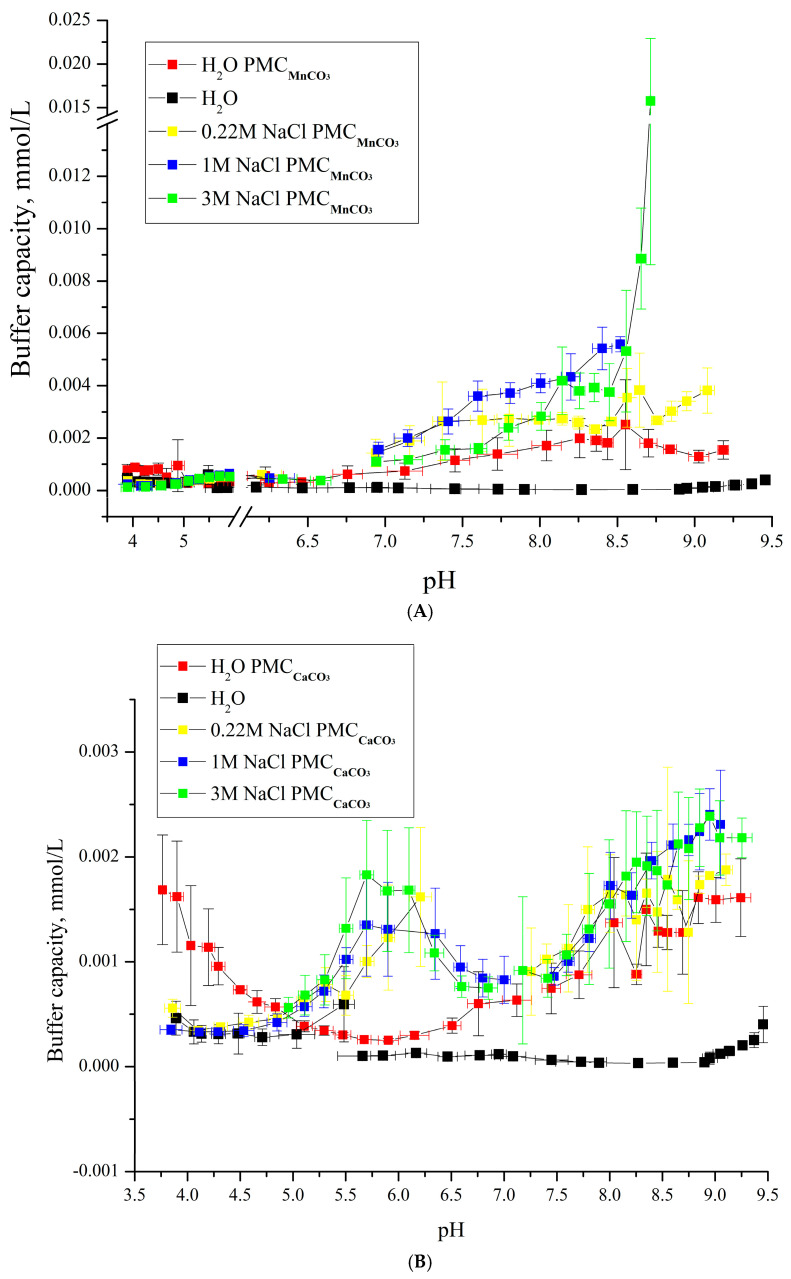
Buffer capacity of PMC composition (PAH/PSS)_3_/PAH at different pH values in water, 0.22 M, 1 M, and 3 M NaCl solutions. (**A**) PMC_Mn_; (**B**) PMC_Ca_ Adapted from [6].

However, to draw definitive conclusions, it is critical to investigate the effects of ionic strength and temperature on the BC of PMC_Mn_, as these factors influence interpolyelectrolyte interactions [36,37]. In the first stage, we analyzed the impact of ionic strength on the BC of PMC_Mn_ within the pH range of 6.5–9. The results are shown in Figure 6.

As shown in Figure 6A,B, the buffer capacity (BC) dynamics of PMC_Mn_ and PMC_Ca_ differ in acidic pH ranges: at pH < 5, the BC of PMC_Ca_ decreases, while that of PMC_Mn_ remains unchanged. Under alkaline conditions, the BC behavior of PMC_Mn_ and PMC_Ca_ also diverges. For PMC_Ca_, BC remains consistent across ionic strengths (0.22–3 M NaCl) in the pH range of 6.5–8, but gradually increases at pH > 8, with similar enhancement trends at 1 M and 3 M NaCl. In contrast, PMC_Mn_ exhibits BC growth with increasing NaCl concentration up to 1 M. At 3 M NaCl, a sharp BC increase occurs at pH > 8.5, a behavior characteristic of PMC_PS_. The sharp BC surge at 3 M NaCl (Figure 6A) exemplifies threshold-driven nonlinearity, where ionic screening collapses electrostatic barriers, enabling rapid shell reorganization—analogous to percolation transitions in Sun’s colloidal wave model [38]. This finding challenges the initial hypothesis, suggesting that PMC_Mn_’s BC dynamics may vary not only with ionic strength but also under temperature changes.

**Figure 7:**
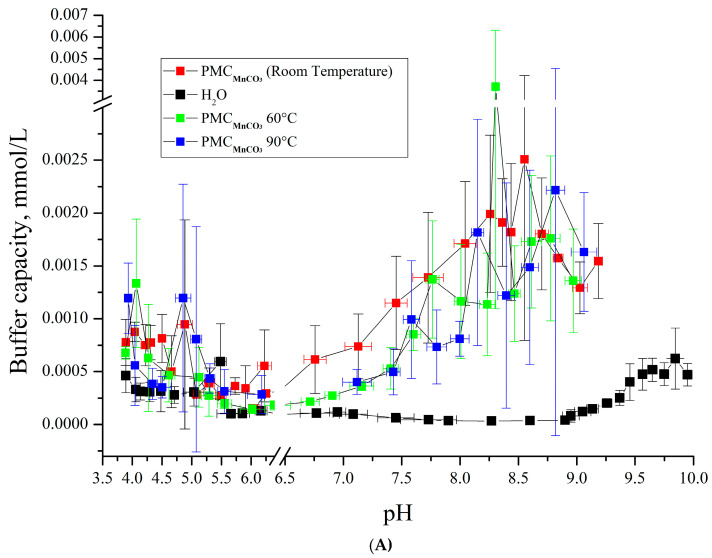

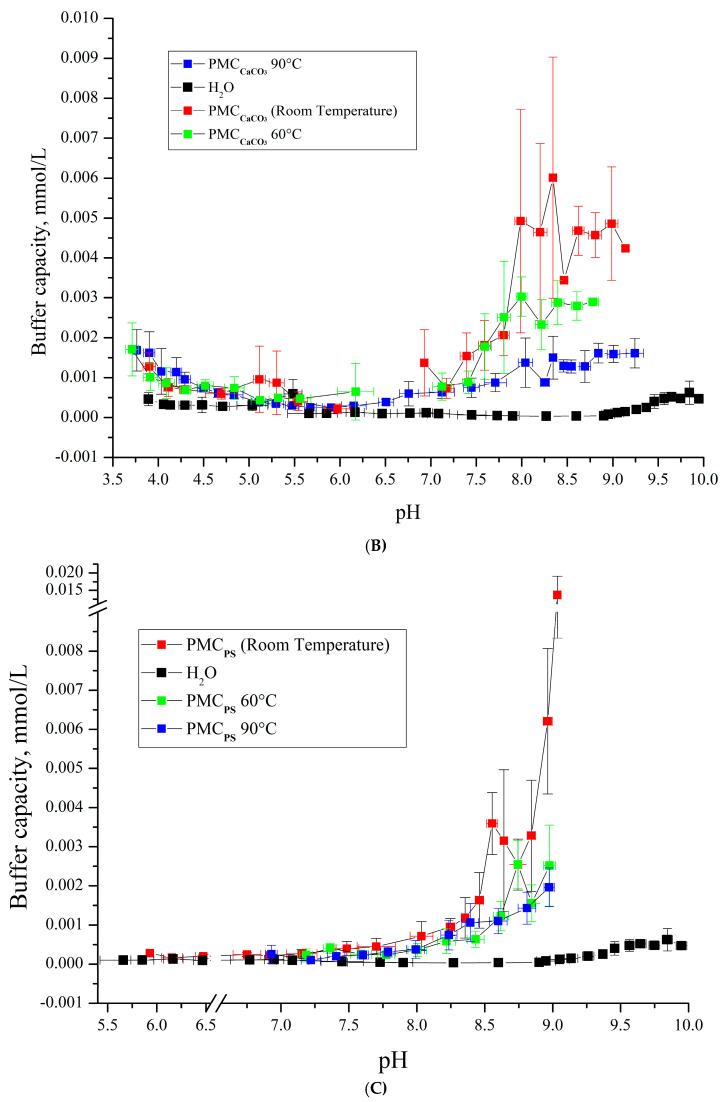
Buffer capacity of PMC composition (PAH/PSS)_3_/PAH at different pH in water, 0.22 M, 1 M, and 3 M NaCl solution. (**A**) PMC_Mn_; (**B**) PMC_CaCO3_ Adapted from [6]; (**C**) PMC_Ps_ Adapted from [20].

In the next stage, we investigated the effect of temperature on the BC of PMC_Mn_ within the pH range of 6.5–9. The results are presented in Figure 7.

As shown in Figure 7A,B, the buffer capacity (BC) dynamics of PMC_Mn_ and PMC_Ca_ align under acidic pH conditions. However, their BC profiles diverge in alkaline pH ranges: PMC_Ca_ exhibits a gradual decline in BC with increasing incubation temperature, while PMC_Mn_ remains thermally stable. This BC behavior under heating parallels that of PMC_PS_ (Figure 7C). Notably, at pH > 9, PMC_PS_ retains its BC without thermal treatment, whereas PMC_Mn_ shows no temperature-dependent BC variation. For clarity, the key findings are summarized in Table 1.

Based on the results presented above (Table 1), it can be unequivocally concluded that the use of carbonate cores (MnCO_3_ and CaCO_3_) leads to distinct models of buffer capacity (BC) dynamics in polyelectrolyte microcapsules (PMCs). For instance, the presence of residual ions from the carbonate core, which influence the microcapsule microenvironment, has been discussed in studies of polyelectrolyte capsules templated on CaCO_3_ and MnCO_3_ particles, including their dissolution and ion release during core removal [23,25,36,39]. Specifically, Mn^2+^ ions form stronger complexes with polyelectrolyte functional groups, altering protonation equilibria and BC [40,41].

Furthermore, it can be hypothesized that BC also depends on the spatial orientation of polyelectrolyte layers within the PMCs. Unlike PMC_Ca_, PMC_PS_ and PMC_Mn_ possess an internal cavity unfilled by interpolyelectrolyte complexes. Additionally, the PAH/PSS shells of PMC_PS_ and PMC_Mn_ exhibit greater charge compensation due to their significantly thinner shell structure compared to PMC_Ca_ [24,42,43,44].

## 4. Conclusions

This study demonstrates that polyelectrolyte microcapsules (PMCs) synthesized on manganese carbonate (MnCO_3_) cores exhibit unique hybrid buffering properties, combining characteristics of both CaCO_3_- and polystyrene (PS)-templated microcapsules. It was established that in acidic environments (pH < 5), the buffer capacity (BC) of MnCO_3_-based PMCs resembles that of CaCO_3_-based analogs, attributed to the electrostatic stabilization of poly(allylamine hydrochloride) (PAH) amine groups by Mn^2+^ ions. Under alkaline conditions (pH > 8), their behavior aligns with PS-based PMCs, showing a sharp BC increase at high ionic strength (3 M NaCl), driven by morphological changes in the shell and interpolyelectrolyte interactions. Furthermore, MnCO_3_-based PMCs retain BC across a wide temperature range (60–90 °C), unlike CaCO_3_-based capsules, where heating reduces BC. This thermal stability underscores the role of the core’s chemical nature: Mn^2+^ ions form stronger complexes with polyelectrolytes, reinforcing shell integrity.

These findings broaden the potential applications of MnCO_3_-based PMCs in biomedicine and materials science, particularly in drug delivery systems and microsensors requiring precise pH control under variable ionic strength and temperature. The study confirms that core material selection is a critical factor in designing functional polyelectrolyte microcapsules, paving the way for their optimization for specific applications.

## Figures and Tables

**Table 1:** Comparison of buffer capacity dynamics in polyelectrolyte microcapsules templated on different core materials.

Parameter	PMC_Ca_ (CaCO_3_-core)	PMC_PS_ (PS-core)	PMC_Mn_ (MnCO_3_-core)
BC Dynamics (vs. water)	- pH > 6.2: BC increases - pH < 5: BC increases	- pH > 7: BC increases	- pH > 6.2: BC increases - pH < 5: BC increases
Effect of Ionic Strength (0.22, 1 and 3 M NaCl)	- pH < 5: BC decreases uniformly—pH > 8: BC increases with NaCl concentration up to 1 M	- pH < 5: BC remains unchanged—pH > 8: BC increases with NaCl concentration	- pH < 5: BC remains unchanged—pH > 8.5: BC increases only at 3 M NaCl
Effect of Temperature (60–90 °C)	- pH > 7: BC gradually decreases with heating	- pH > 9: BC decreases at temperatures up to 60 °C	- Entire range (4–10): BC remains unchanged under heating

## Data Availability

Data is contained within the article.
